# Validity and Reliability of the Adolescent Lifestyle Profile-Revised 2 (ALP-R2) Scale in Colombian Adolescents

**DOI:** 10.17533/udea.iee.v42n3e07

**Published:** 2024-10-19

**Authors:** Eugenia del Pilar Herrera Guerra, Lili Rosa Bautista Arellanos, Claudia Bonilla Ibáñez

**Affiliations:** 1 . Nurse, Ph.D. Professor. Universidad de Córdoba; Colombia. Email: edherrera@correo.unicordoba.edu.co. Corresponding author. > https://orcid.org/0000-0001-8413-4935 Universidad de Córdoba Universidad de Córdoba Colombia edherrera@correo.unicordoba.edu.co; 2 . Statistician, Master’s. Professor. Universidad de Córdoba; Colombia. E-mail: lbautistaarellano72@correo.unicordoba.edu.co. https://orcid.org/0000-0002-3353-2244 Universidad de Córdoba Universidad de Córdoba Colombia lbautistaarellano72@correo.unicordoba.edu.co; 3 . Nurse, Ph.D. Professor. Universidad del Tolima, Colombia. Email: cbonilla@ut.edu.co. https://orcid.org/0000-0003-2525-4798 Universidad del Tolima Universidad del Tolima Colombia cbonilla@ut.edu.co

**Keywords:** adolescent, Health promotion, Psychometrics, Methodological research in nursing, Surveys and questionnaires., adolescente, promoción de la salud, Psicometría, Investigación metodológica en enfermería, Encuestas y cuestionarios., adolescente, Promoção de saúde, Psicometria, Pesquisa metodológica em enfermagem, Pesquisas e questionários.

## Abstract

**Objective.:**

The study sought to determine the validity and reliability of the Adolescent Lifestyle Profile-Revised 2 (ALP-R2) scale, translated into Spanish, in Colombian adolescents.

**Methods.:**

Psychometric study, which included the translation process (English to Spanish). The final version of the scale in Spanish was approved by Nola Pender; apparent and content validation was carried out through expert judgment (*n* = 6). With a sample of 1476 Colombian adolescents. Construct validity was determined through exploratory and confirmatory factor analysis. The internal consistency was calculated with Cronbach’s coefficient.

**Results.:**

Favorable evidence was obtained of apparent validity, content validity, and construct validity with a model comprised of 44 items and 7 subscales (total accumulated variance 44.9%) with good absolute fit (χ*2*: 18434.3; *df* = 946; *p* < 0.0001; CMIN/DF = 4.326; SRMR = 0.0562; RMSEA = 0.047), incremental (CFI = 0.834; NFI = 0.795; NNFI = 0.822) and parsimony (PCFI = 0.777; PNFI = 0.741; AIC = 4116.848). Good internal consistency in the total scale (α = 0.91) and in the subscales (α between 0.609 and 0.809).

**Conclusion.:**

The Spanish version of the ALP-R2 scale has adequate psychometric properties of validity and reliability, to measure the lifestyle profile of Colombian adolescents, coherent with the theoretical model of Health Promotion. Its use is recommended in populations similar to that of the study.

## Resumen

**Objetivo.:**

Determinar la validez y confiabilidad de la escala Adolescent Lifestyle Profile-Revised 2 (ALP-R2) traducida al español, en adolescentes colombianos.

**Métodos.:**

Estudio Psicométrico, que incluyó el proceso de traducción (inglés a español). La versión final de la escala en español fue aprobada por Nola Pender, se le realizó validación aparente y de contenido mediante juicio de expertos (*n* = 6). Con una muestra de 1.476 adolescentes colombianos. Se determinó la validez de constructo mediante análisis factorial exploratorio y confirmatorio. La consistencia interna se calculó con el coeficiente alfa de Cronbach.

**Resultados.:**

Se obtuvo evidencia favorable de validez aparente, de validez contenido y de validez de constructo con un modelo conformado por 44 ítems y 7 subescalas (varianza total acumulada 44,9 %) con buen ajuste absoluto (χ*2*: 18434.3; *gl* = 946; *p* < 0.0001; CMIN/DF = 4.326; SRMR = 0.0562; RMSEA = 0.047), incremental (CFI = 0.834; NFI = 0,795; NNFI = 0,822) y de parsimonia (PCFI = 0,777; PNFI = 0,741; AIC = 4116.848). Buena consistencia interna en la escala total (α = 0.91) y en las subescalas (α entre 0.609 y 0.809).

**Conclusión.:**

La escala ALP-R2 versión español tiene adecuadas propiedades psicométricas de validez y confiabilidad, para la medición del perfil estilo de vida de los adolescentes colombianos, coherente con el Modelo teórico de Promoción de la Salud. Se recomienda su uso en poblaciones similares a la del estudio.

## Resumo

**Objetivo.:**

Determine a validade e confiabilidade da escala Adolescent Lifestyle Profile-Revised 2. (ALP-R2) traduzido para o espanhol, em adolescentes colombianos.

**Métodos.:**

Estudo Psicométrico, que incluiu o processo de tradução (inglês para espanhol). A versão final da escala em espanhol foi aprovada por Nola Pender e a validação de conteúdo foi realizada por meio de parecer de especialistas (*n* = 6). Com uma amostra de 1.476 adolescentes colombianos. A validade de construto foi determinada por meio de análise fatorial exploratória e confirmatória. A consistência interna foi calculada através do coeficiente alfa de Cronbach.

**Resultados.:**

Evidências favoráveis de validade aparente, validade de conteúdo e validade de construto foram obtidas com um modelo composto por 44 itens e 7 subescalas (variância total acumulada 44.9%) com bom ajuste absoluto (χ *2*: 18434.3; *gl* = 946; *p*< 0.0001; CMIN /DF = 4.326; Boa consistência interna na escala total (α = 0.91) e nas subescalas (α entre 0.609 e 0.809).

**Conclusão.:**

A escala ALP-R2, versão em espanhol, possui propriedades psicométricas adequadas de validade e confiabilidade para medir o perfil de estilo de vida de adolescentes colombianos, consistentes com o Modelo teórico de Promoção da Saúde. Seu uso é recomendado em populações semelhantes à do estudo.

## Introduction

According to the World Health Organization (WHO), adolescence is a stage of life (10 to 19 years) where behavioral patterns related to lifestyle are established, which can be protective or risky in the present and in the future.^1^ The concept of lifestyle has been defined as an overall way of life, based on the interaction between living conditions and individual behavioral patterns, determined by sociocultural factors and personal characteristics;^2^ while the lifestyle profile related with health is understood as a set of activities with significant influence on the state of health and are a regular part of the daily pattern of a person's life.^3^ In adolescents, the lifestyle profile is defined through behaviors that promote health, such as physical activity, positive outlook on life, interpersonal relationships, health responsibility, stress management, and spiritual health.^4^ Scientific evidence indicates that an unhealthy lifestyle is the sum of behavioral risk factors (physical inactivity, unhealthy diet) which lead to the development of cardiovascular disease and diabetes among other chronic non-communicable diseases (CNCD).^5^^-^^7^

The CNCD are a public health concern of major relevance globally and represent an economic burden for health services. It is estimated that by 2030, the proportion of deaths due to CNCD will reach 70% and the global morbidity burden will reach 56%.^8^ Meanwhile, in Colombia in recent years CNCD register a considerable increase in the rate of care per 100-thousand inhabitants, as consequence of their high incidence. Due to the foregoing, it is estimated that health costs will increase by nearly 40% between 2022 and 2030.^9^ The high incidence of CNCD is associated with modifiable behavioral risk factors, like physical inactivity, unhealthy diet, and tobacco and alcohol consumption. Studies have revealed the benefits of a healthy lifestyle in preventing and managing CNCD during all life stages.^10^^,^^11^

In this regard, the WHO considers that adolescence is an important moment of human development to lay the foundations for good health.^1^ Nursing professionals are the largest segment of health professionals and are in a key position to assume leadership for health promotion, helping people from all ages to stay healthy and create healthy environments, with a holistic approach, from an individual perspective. Nurses assess the health-promoting lifestyle profile, as base to adapt a health promotion plan and to make clinical decisions. This assessment also shapes the nature of the client-nurse partnership, such as frequency of contact and the need for coordination with other health professionals.^12^ The aforementioned highlights the need to evaluate healthy lifestyles in adolescents to be able to influence the primary prevention of CNCD by implementing health promotion and maintenance interventions, which are effective in achieving changes related with behavioral risk factors. However, for researchers, academics, and health professionals to be able to evaluate the health-promoting lifestyles of adolescents (baseline) and to objectively demonstrate the changes in them after receiving an intervention, valid and reliable measurement instruments are required.

The most commonly used instruments to assess the health-promoting lifestyle in adolescents are: the Fantastic^13^ and the Adolescent Lifestyle Profile - Revised 2 (ALP-R2) scale; the latter is designed for adolescents. In its original version, it has evidence of construct validity with a seven-factor structure and reliability tests with a Cronbach’s alpha of 0.93.^4^ Notwithstanding, said scale has not been translated and validated in the Spanish version, which limits its use in Colombian adolescents, given that its psychometric properties are unknown. The aim of this study was to determine the validity and reliability of the ALP-R2 scale translated into Spanish in Colombian adolescents, so that it can be used in nursing research and practice and by other professionals interested in evaluating lifestyles and proposing effective interventions to promote a health-promoting behavior in adolescents.

## Methods

A psychometric study was used, which included the translation process and adaptation of the ALP-R2 scale (English to Spanish) as previous phase, and the tests of apparent validity, content validity, construct validity, and reliability for the Spanish version.

### Instrument

The ALP-R2 scale was developed from the Health-Promoting Lifestyle Profile II scale designed with the Health Promotion Model (HPM) by Nola Pender for adult population. The study by Hendricks *et al*., ^(^^4^ modified the initial 42-item scale, adding two items and dividing the personal growth subscale in two: spiritual health and positive outlook on life, resulting in the ALP-R2 scale made up of 44 items and 7 subscales: health responsibility (items: 3, 8, 14, 22, 33, 34, 44), physical activity (items: 2, 4, 16, 27, 32, 40), nutrition (items: 7, 10, 13, 21, 24, 30, 42) positive outlook on life (items: 18, 23, 26, 28, 38, 39), interpersonal relationships (items: 1,6, 12, 19, 31, 37), stress management (items 5, 11, 17, 25, 36, 43), and spiritual health (items: 9, 15, 20. 29, 35, 41). The ALP-R2 scale uses a 4-point Likert-type response format (1: never, 2: sometimes, 3: often, and 4: always). The score can be obtained in both the subscales as from the total scale by adding the items, for the total scale being a minimum value of 44 and a maximum of 176. The higher the score, the better the health-promoting lifestyle profile behaviors of adolescents.^4^

### Procedure

The translation of the ALP-R2 scale from English to Spanish was carried out independently by two bilingual translators (a nurse with experience on the construct to measure and an official translator without knowledge on the theme). Both translations were evaluated considering the semantic and cultural equivalence between the original version and the one translated by a panel of experts (researchers, two nurses, and a linguist). The back-translation from Spanish to English was carried out independently by two new native English translators. The translation into Spanish and the backtranslation were approved by Dr. Nola Pender. The evaluation of the apparent and content validity of the Spanish version of the ALP-R2 scale was performed by a group of six Colombian nurses, considered experts given their experience in psychometric studies and professional formation. 

To conduct the construct validity tests, a random sample representative of the total population of 1,476 adolescents was used, calculated with a 95% confidence level (standard error = 5%; *p* = 0.5; q = 0.5; Kish index = 0.15). The recommended sample size for factor analysis was also considered by the number of absolute cases (>200) and cases per observed variable, which considers the sample size according to the number of indicators and the number of latent constructs, suggesting 15 cases per observed variable. ^(^^14^ The sample was selected through convenience, to include both phases of adolescence: early (10-14) and late (15-19). Students enrolled in four public and private educational institutions in the city of Ibagué-Colombia, aged 10 to 19 years, were included. The study excluded adolescents who were diagnosed with chronic diseases and/or physical limitations that impeded engaging in physical activity and/or who had special diets; information obtained by self-report. Each of the adolescents completed the Spanish version of the ALP-R2 scale and the sociodemographic form was developed by the researchers, with prior consent from the adolescents and informed consent from the parents. To ensure data quality, training was provided to research assistants in the application of the instrument, verification of the completion of the entire scale, and correct completion of the study database.

### Data analysis

To evaluate the apparent and content validity of the ALP-R2 scale, Fleiss’s kappa index was used and the content validity ratio and content validity index were calculated according to the modified Lawshe model.^15^ In the construct validity tests of the ALP-R2 scale a descriptive analysis was carried out of the data (mean, standard deviation, asymmetry, kurtosis, and item-total corrected correlation coefficient). Adequacy of the sample size and the correlation among the variables was evaluated through the Kaiser-Meyer-Olkin test (≥ 0.6 is acceptable) and Bartlett's test of sphericity (*p* <0.05). To assess the contribution by each item to its respective subscale, factor loadings were calculated through an exploratory factor analysis (EFA), using the principal component analysis extraction method and Oblimin rotation with Kaiser normalization. 

For the confirmatory factor analysis (CFA), measures of the model’s fit to the data were calculated based on the relationship between the Chi-square value (χ^2^) and the degrees of freedom (df). It was considered that the values of χ^2^ / df < 5 and the cut-off points recommended for each of the measures of absolute fit (Chi-square weighted by degrees of freedom, standardized mean square error, root mean square error of approximation); measures of incremental fit (comparative fit index, normed fit index and non-normed fit index) and measures of parsimony fit (comparative fit index de parsimony, parsimony normed fit index and Akaike information criterion.(^16^) Reliability was evaluated using internal consistency as criterion by calculating Cronbach’s coefficient (α >0.7). Data was analyzed in SPSS 22 and AMOS 22 statistical software.

Ethical aspects. This study was approved by the ethics committee at Universidad del Tolima and was authorized by the rectors of the educational institutions where information was collected. All participants had informed consent signed by their parents and the assent of minors. International and national ethical guidelines were followed (Resolution 8430 of 1993 and Legislation 911 of 2004). 

## Results

### Apparent and content validity

The 44 items in the Spanish version of the ALP-R2 scale were rated by the panel of experts (*n* = 6). Substantial agreement was obtained (Fleiss Kappa index of 0.8 in comprehension, 0.8 in clarity and 0.8 in precision). All the items were accepted (content validity ratio > 0.8) with satisfactory content validity index (0.9). 

### Construct validity

Of the entire study sample (*n* = 1,476) more men (53.5%) participated than women. The minimum age was 10 years and the maximum age was 19 years, with a mean of 13.8±1.8 years, with the highest frequency in the group from 12 to 16 years of age (81.9%). It was found that 73.1% was studying basic secondary educational level and the remaining 26.9% was studying in primary education. 

### Descriptive statistics and correlations among the items of the ALP-R2 scale

Table 2 shows that the average scores of the items **in the** ALP-R2 scale**were between** 1.6±0.82 and 3.47±0.95, obtaining the lowest scores in items 33 and 22 and the highest scores in item 44 of the health responsibility subscale and in items 13 and 30 of the nutrition subscale. The data distribution in the items was approximately symmetric, recording symmetry values ​​between -1.58 and 1.40 and kurtosis between -1.38 and 1.39, from which normality is inferred. The internal consistency of the ALP-R2 total scale (α = 0.913) and for each of the subscales resulted reliable. 

According with the EFA results (KMO = 0.931; χ*2*: 18434.331; *df* = 946; *p*<0.0001), the item-total correlation maintains a 7-factor structure and the location of the items in the seven subscales, as contemplated in the theoretical model of the original version of the ALP-R2 scale** **with an accumulated variance of 44.9% and α = 0.913 (95% CI: 0.906 - 0.919). Now, the reliability analysis suggests eliminating item 44, bearing in mind the predefined criterion (item-total correlations < 0.2). Since a value far from the reference measure is not recorded, in addition to not being negative and noting that the increase in the alpha values ​​is not large, the exclusion of said item is not considered, however, the need to study the suggested model is created. Regarding the contribution of the items within each subscale to the variation of the responses given by the Colombian adolescents participating in the study, with the EFA it can be seen that for the health responsibility subscale the lowest variation was registered in item 44; in physical activity in item 2; in nutrition in items 13 and 30 followed by items 10 and 42; in positive outlook on life in item 26; in interpersonal relationships in items 1 and 19; and in stress management in item 17. In spiritual health, all the items have large significant contribution (> 0.5) indicating the higher factor loading in item 41. Moreover, the highest variation was found in items 34, 16, 7, 18, 12, and 11, as shown in Table 1.

**Table 1:** Descriptive statistics, correlations between the items and factor loading (EFA)

Items	Mean ± SD^a^	Asymmetry	Kurtosis	Item-total Correlation	α^b^ if the item is suppressed	Factor loading
Health responsibility (α = 0.694; 95% CI: 0.669 - 0.717) % Variance by factor 2.4%						
3	1.88 ± 0.97	0.980	0.004	0.439	0.650	0.640
8	1.84 ± 0.72	0.735	0.749	0.343	0.675	0.373
14	2.26 ± 0.98	0.451	-0.764	0.518	0.626	0.671
22	1.68 ± 0.84	1.178	0.779	0.411	0.658	0.609
33	1.60 ± 0.82	1.401	1.390	0.466	0.645	0.710
34	2.03 ± 0.94	0.715	-0.329	0.576	0.609	0.774^‡^
44	3.47 ± 0.95	-1.581	1.083	0.198**	0.737	0.083^†^
Physical activity (α = 0.809; 95% CI: 0.793 - 0.824) % Variance by factor 6.1%						
2	2.53 ± 0.85	0.321	-0.660	0.307	0.828	0.282^†^
4	2.8 0± 1.04	-0.214	-1.228	0.636	0.763	0.795
16	2.98 ± 1,01	-0.411	-1.151	0.667	0.756	0.803^‡^
27	2.84 ± 0.96	-0.163	-1.150	0.589	0.775	0.653
32	2.97 ± 1,01	-0.430	-1.091	0.604	0.771	0.736
40	2.83 ± 1,03	-0.244	-1.210	0.602	0.771	0.762
Nutrition (α = 0.609; 95% CI: 0.578 - 0.639) % Variance by factor 4.6%						
7	2.03 ± 0.74	0.699	0.694	0.250	0.594	0.673^‡^
10	2.45 ± 0.95	0.234	-0.863	0.298	0.581	0.267^†^
13	3.47 ± 0.86	-1.368	0.539	0.254	0.594	0.027^†^
21	2.37 ± 0.87	0.443	-0.467	0.479	0.517	0.366
24	2.42 ± 0.90	0.347	-0.678	0.391	0.547	0.490
30	3.47 ± 0.73	-1.111	0.229	0.252	0.593	0.029^†^
42	2.85 ± 1.01	-0.277	-1.145	0.345	0.565	0.270^†^
Positive outlook on life (α = 0.754; 95% CI: 0.734 - 0.773) % Variance by factor 22.0%						
18	2.97 ± 0.97	-0.383	-1,079	0.548	0.703	0.679^‡^
23	3.25 ± 0.93	-0.856	-0.549	0.554	0.702	0.641
26	3.25 ± 0.84	-0.785	-0.373	0.206	0.787	0.372^†^
28	2.78 ± 0.98	-0.077	-1,200	0.595	0.689	0.676
38	3.29 ± 0.82	-0.794	-0.436	0.555	0.704	0.643
39	3.44 ± 0.81	-1,220	0.425	0.524	0.712	0.669
Interpersonal relationships (α = 0.627; 95% CI: 0.597 - 0.656) % Variance by factor 3.4%						
1	2.92 ± 0.83	-0.101	-1.024	0.285	0.610	0.173^†^
6	3.04 ± 0.89	-0.378	-1.005	0.433	0.554	0.692
12	2.68 ± 0.97	-0.043	-1.068	0.385	0.573	0.700^‡^
19	2.95 ± 0.91	-0.380	-0.831	0.238	0.629	0.207^†^
31	2.90 ± 0.91	-0.169	-1.118	0.363	0.582	0.514
37	3.04 ± 0.90	-0.481	-0.767	0.451	0.547	0.637
Stress management (α = 0.691; 95% CI: 0.665 - 0.714) % Variance by factor 2.5%						
5	3.12 ± 0.88	-0.465	-0.993	0.395	0.658	0.345
11	2.74 ± 0.91	0.010	-1.038	0.442	0.643	0.720^‡^
17	2.80 ± 0.90	-0.005	-1.102	0.298	0.688	0.262^†^
25	3.20 ± 0.82	-0.515	-0.983	0.501	0.627	0.581
36	2.82 ± 0.96	-0.200	-1.073	0.481	0.629	0.309
43	2.51 ± 1.01	0.127	-1.094	0.419	0.652	0.349
Spiritual health (α = 0.788; 95% CI: 0.771 - 0.805) % Variance by factor 4.0%						
9	1.73 ± 0.81	1.052	0.713	0.424	0.781	0.540
15	2.62 ± 1.12	-0.036	-1.384	0.399	0.795	0.524
20	1.73 ± 0.96	1.148	0.228	0.519	0.761	0.733
29	2.06 ± 0.96	0.637	-0.517	0.645	0.730	0.758
35	2.22 ± 0.97	0.465	-0.723	0.594	0.743	0.727
41	2.17 ± 1.01	0.478	-0.848	0.680	0.719	0.794^‡^

Extraction method: principal components analysis; Rotation method: Oblimin with Kaiser normalization; χ*2*: chi-squared; *df*: degrees of freedom; *significance *p* > 0.05; a: Standard deviation; b: Cronbach’s alpha; 95% CI: 95% Confidence Interval for Cronbach’s alpha; ** Item-total correlation <0.2; ^‡^ item that contributes the most to the variation; ^†^ item that contributes the least to the variation.

Table 2 shows that the mean score of the total ALP-R2 was 2.66 ± 0.42. Among the subscales, higher scores were obtained in positive outlook on life (3.16 ± 0.60) and the lowest scores were for health responsibility (1.88 ± 0.58). According to sex, the scores of the total APL-R2 and the subscales differed between men and women. Overall, men had higher scores, thereby, better health-promoting behavior compared with women (2.75 ± 0.39 against 2.56 ± 0.43; t = 8.716, *p* <0.001). Likewise, significant differences were found among the scores of the subscales, except for the interpersonal relationship’s subscale (t = 0.796, *p* = 0.426).

**Table 2:** Cronbach’s alpha and comparison of means of the suggested model according to sex

Subscale	Cronbach’s Alpha	General sample	Men (*n* = 789)	Women (*n* = 687)	***p (*t value)**
RH	0.737	1.88 ± 0.58	1.92 ± 0.60	1.83 ± 0.56	0.005^**^ (t = 2.845)
PA	0.809	2.82 ± 0.70	3.03 ± 0.65	2.59 ± 0.69	<0.001^*^ (t = 12.718)
N	0.609	2.72 ± 0.48	2.81 ± 0.45	2.61 ± 0.49	<0.001^**^ (t = 8.15)
POL	0.754	3.16 ± 0.60	3.29 ± 0.54	3.02 ± 0.62	<0.001^**^ (t = 9.017)
IR	0.627	2.92 ± 0.53	2.93 ± 0.52	2.91 ± 0.55	0.426 (t = 0.796)
SM	0.691	2.86 ± 0.57	2.98 ± 0.53	2.73 ± 0.60	<0.001^**^ (t = 8.316)
SH	0.788	2.09 ± 0.68	2.12 ± 0.69	2.05 ± 0.66	0.044^*^ (t = 2.02)
TOTAL	0.913	2.66 ± 0.42	2.75 ± 0.39	2.56 ± 0.43	<0.001^**^ (t = 8.716)

RH = health responsibility; PA = physical activity; N = nutrition; POL = positive outlook on life; IR = interpersonal relationships; SM= stress management; SH= spiritual health; Mean ± Standard deviation; t: Student’s t-statistic; *p= p* value ^**^ The difference is significant at 0.01 level (*p* < 0.01); ^*^ The difference is significant at 0.05 level (*p* < 0.05).

### Construct validity

Table 3 shows that the CFA results provide satisfactory adjustment indices for both the 44-item theoretical model (CMIN/DF = 4.326, SRMR = 0.0562, RMSEA = 0.047) and for the 43-item suggested model (CMIN/DF = 4.367, SRMR = 0.0558, RMSEA = 0.048); considering that the measure of absolute fit that determines the degree to which the general model predicts the correlation matrix met the minimum necessary requirements (CMIN/DF < 5), similar to the measure that represents the anticipated adjustment with the total value of the population (RMSEA < 0.08) and the measure that measures the standardized residual covariance of the sample (SRMR < 0.08). With respect to the measures of incremental fit that allow comparing the improvement in the fit of the suggested model in relation with a theoretical model, satisfactory results were obtained given that in each of the indices evaluated an increase was registered for the suggested model (CFI = 0.839, NFI = 0.801, NNFI = 0.827) against the results in the base model (CFI = 0.834, NFI = 0.795, NNFI = 0.822), approaching the recommended threshold in each case (> 0.90).

Both models under study were compared by analyzing the measures of parsimony fit that relate the constructs with the theory that supports them and when considering the degrees of freedom available in the measures of incremental fit, these turn out to be indices less sensitive to the sample size. The indices of the suggested model (PCFI = 0.780. PNFI = 0.745, AIC = 3964.080) contrasted with the indices of the theoretical model (PCFI = 0.777, PNFI = 0.741, AIC = 4116.949) were better; being adequate in both cases (PCFI, PNFI > 0.5), it was noted that when excluding item 44 the PCFI and PNFI indices increased getting closer to 1.0, indicating a better fit in the model with respect to the relation of the items with the subscale they seek to explain. In addition, upon analyzing the comparative measures of adequacy with the Akaike information criterion (AIC) as the smaller values indicate a better fit, the suggested model was considered the most adequate. Although the suggested model provides some improvement in the adjustment indices regarding the theoretical model, it must be highlighted that said improvement is minimal, which confirms that not much is gained by considering the exclusion of item 44, however, a review is suggested in the revision of this item in similar populations. 

**Table 3:** Confirmatory factor analysis (CFA)

Adjustment indices	Recommended cut-off points	Theoretical model (44 items)	Suggested model (43 items)
Measures of absolute fit			
CMIN/DF^a^	< 5	4.326	4.367
SRMR^b^	< 0.08	0.0562	0.0558
RMSEA^c^	< 0.08	0.047	0.048
CI^d^ 90% RMSEA		0.046 - 0.049	0.046 - 0.049
Measures of incremental fit			
CFI^e^	> 0.90	0.834	0.839
NFI^f^	> 0.90	0.795	0.801
NNFI^g^	> 0.90	0.822	0.827
Measures of parsimony fit			
PCFI^h^	> 0.5	0.777	0.780
PNFI^i^	> 0.5	0.741	0.745
AIC^j^	The smaller the better	4116.949	3964.080

Source: elaborated by the authors. a: Chi-square weighted by degrees of freedom, b: standardized mean square error, c: root mean square error of approximation, d: confidence interval, e: comparative fit index, f: normed fit index, g: non-normed fit index, h: comparative parsimony fit index, i: parsimony normed fit index, j: Akaike information criterion.

**Figure 1:**
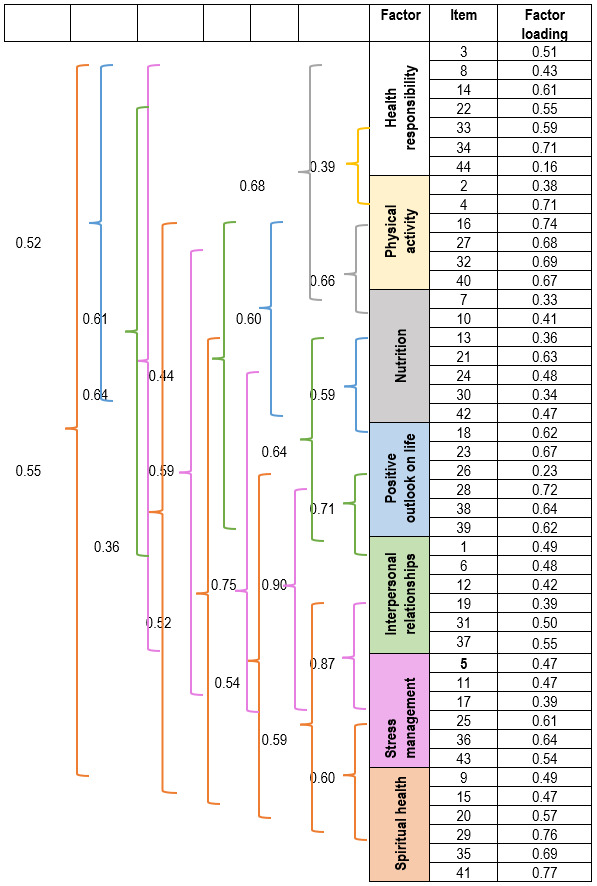
Structural diagram of the confirmed theoretical model and standardized correlations

Bearing in mind the EFA and CFA results, the ALP-R2 was finally comprised of 44 items and 7 dimensions conserving the original theoretical model. The factor structure and correlations obtained between the variables and the items of each of the subscales are shown in Figure 1.

### Reliability

The scale’s reliability was evaluated through internal consistency. Table 2, already shown, evidences that Cronbach’s α coefficients for the total ALP-R2 was of high reliability (α = 0.913). Cronbach’s α values ranged between 0.609 and 0.809. The subscales for physical activity (α = 0.809), positive outlook on life (α = 0.754), stress management (α = 0.691), health responsibility (α = 0.694), and spiritual health (α = 0.788) were acceptable. Questionable values were found (< 0.7) in the subscales for nutrition α = 0.609 (95% CI = 0.578 - 0.639) and interpersonal relationships α = 0.627 (95% CI = 0.597 - 0.656), however, with a 95% confidence level, it is estimated that the value is close to 0.70 and does not reach unacceptable scales (< 0.50).

Table 4 shows that the correlations among the subscales were statistically significant (*p* < 0.01) from moderate to strong, ranging between 0.307 (Spiritual health - Physical activity) and 0.646 (Stress management - Positive outlook on life). All the subscales showed correlations with the total scale that ranged between 0.677 (Spiritual health - Total) and 0.766 (Positive outlook on life - Total), being significant from moderate to strong. In general, all the correlations registered were positive.

**Table 4:** Pearson’s correlations between factors from the theoretical model and the total score

	RH r^a^	PA r	N r	POL r	IR r	SM r	SH r
RH	1						
PA	0.337^**^	1					
N	0.467^**^	0.506^**^	1				
POL	0.388^**^	0.509^**^	0.434^**^	1			
IR	0.424^**^	0.357^**^	0.420^**^	0.489^**^	1		
SM	0.464^**^	0.463^**^	0.512^**^	0.646^**^	0.583^**^	1	
SH	0.430^**^	0.307^**^	0.356^**^	0.422^**^	0.427^**^	0.438^**^	1
TOTAL	0.683^**^	0.700^**^	0.720^**^	0.766^**^	0.716^**^	0.803^**^	0.677^**^

RH: Health responsibility, PA: Physical activity, N: Nutrition, POL: Positive outlook on life, IR: Interpersonal relationships, SM: Stress management, SH: Spiritual health. a: Pearson’s correlation coefficient. **p < 0.01.

## Discussion

This is the first study exploring the validity and reliability of the Spanish version of the ALP-R2 scale in a sample of Colombian adolescents. The psychometric properties evaluated were adequate, reaffirming that it is a multidimensional scale composed of 44 items and 7 subscales that measure a single construct “lifestyle profile of the adolescent” theoretically supported by the HPM as a health-promoting behavior. According to Hendricks *et al.,*^4^ adolescents establish behavioral patterns and make decisions about their lifestyles that affect their future health during their transition from childhood to adulthood. Therein, the importance of having an instrument like the ALP-R2 that permits measuring integrally seven domains of a lifestyle that promotes health: health responsibility, physical activity, nutrition, positive outlook on life, interpersonal relationships, stress management, and spiritual health.

The ALP-R2 scale has subscales similar to other instruments that also measure lifestyle in adolescents, like the Adolescent Lifestyle Questionnaire (ALQ)^17^ and Health-Promoting Lifestyle Profile scale (HPLP),^18^ but differs from these mainly in that it evaluates the spiritual health dimension and does not include the social support dimension.

The psychometric characteristics of the ALP scale have been revised in adolescents in the late adolescence phase (15 to 19 years of age) in the United States (*n* = 311-15 to 17 years of age),^19^ Brazil (*n* = 236 -14 to 18 years of age)^20^ and in Türkiye (*n* = 890 -14 to 18 years of age).^21^^,^^22^ In Spanish-speaking countries, Gaete *et al.,*^23^ evaluated the validity and reliability of the Spanish version of the de ALP-R2 in Chile (*n* = 572 - 14 to 21 years of age). The dimensionality and reliability of the Colombian Spanish version of the ALP-R2 scale found in this study were equivalent to the factor structure of the original version reported by Hendricks *et al*.,^4^ of 44 items and 7 subscales. Likewise, the total Cronbach's α coefficient (α = 0.93) and of the subscales are similar, reporting values α >0.7 in the subscales of spiritual health (α = 0.82), positive outlook on life (α = 0.81), physical activity (α = 0.77), and α <0.7 in nutrition (α = 0.65) and stress management (α =0.66). Differences were found in health responsibility (α = 0.82) and interpersonal relationships (α = 0.77) with values higher than those in the present study.

The results are also similar to those reported in Chile by Gaete *et al.,*^23^ which also confirmed the theoretical model of 44 items and 7 subscales. The construct validity tests also reported acceptable parameters (SRMR =0.08; GFI =0.98); AGFI =0.97; NNFI =0.97 and RMSEA =0.07; NFI = 0.83 and CFI = 0.87). Nevertheless, the reliability of the global scale (α = 0.87) was lower than that of the original version (α = 0.93) and lower than that found in this study (0.913). Similarly, the reliability of the subscales was lower (α values between 0.49 and 0.85). The lowest value was stress management (α = 0.49) and nutrition (α = 0.55) and the highest value was physical activity (α = 0.85). Furthermore, it was found that all the items had correlations among them and the global scale. Within the health responsibility subscale, item 44 (“I avoid behaviors that are harmful to my health (smoking, alcohol consumption, drug use, sexual activities)) had low correlation with the total scale. This may be explained in part by the fact that adolescents in Colombia do not believe it is a behavior harmful to health. In Colombia, abuse and addiction to psychoactive substances is a public health problem. Consumption of illegal psychoactive substances starts early. The mean onset age is 14.1 years; among the factors that influence consumption in school-aged adolescents are the availability and supply of drugs, low perception of risk in relation to their consumption, and low parental involvement.^24^ Regarding the association analysis by gender, significant differences were found among the scores of the subscales, except for the interpersonal relationships subscale (t = 0.796, *p* = 0.426). These findings differ somewhat with those reported in the Chilean Spanish version of the ALP-R2 where statistically significant differences between men and women were only found in four subscales (health responsibility, interpersonal relationships, spiritual health, and physical activity). 

The Colombian Spanish version of the ALP-R2 scale has acceptable psychometric characteristics, which unlike prior studies were evaluated with a robust sample of Colombian adolescents in early and late adolescence phase, as defined by the WHO (10 to 19 years of age). Consequently, it can allow evaluating interventions that promote healthy behaviors and prevent risk behaviors. Nonetheless, this study has some limitations; it was conducted on a convenience sample from a city in Colombia, hence, it cannot be said to be representative of the entire country. This study did not test the capacity to detect changes over time, therefore, it is recommended to conduct experimental studies that allow evaluating the effect of interventions on the health-promoting behavior of adolescents. 

The importance of the present study could have for the nursing discipline and for the adolescent population, lies mainly in being able to have a valid and reliable instrument that permits integrally evaluating the health promoting lifestyle, which allows it to be used in research to test the effectiveness of nursing interventions designed and validated under the HPM theoretical framework. The impact of the study’s results on improving knowledge and the nursing practice is mainly based on being able to employ a useful, practical, simple, and necessary empirical indicator for comprehensive assessment of behaviors that promote the health of adolescents (health responsibility, physical activity, nutrition, positive outlook on life, interpersonal relationships, stress management, and spiritual health), with potential to guide better interventions, which permit achieving better health results and practical applications that reinforce the levels of evidence of nursing leadership in promoting and maintaining adolescent health.

This study concludes that the tests of content validity, construct validity, and reliability performed on the Spanish version of the ALP-R2 scale indicated that the scale is valid and reliable and can be used to evaluate lifestyles in adolescents. Further studies are needed to explore the psychometric properties of the ALP-R2 in other Spanish-speaking countries in Latin America to assess the possible cultural differences.

## Annex. Spanish version of the Adolescent Lifestyle Profile and its equivalent in English of the Adolescent Lifestyle Profile-Revised 2

Instructions: reflect carefully on each of the following statements in this questionnaire, and tell us how often you display each of these behaviors by marking with an X in the corresponding box:

Response options: 1: never, 2: sometimes, 3: often, 4: always.

**Table:**

Item	In Spanish	In English
1	Paso tiempo conversando con miembros de mi familia.	I spend time speaking with my relatives.
2	Paso tiempo realizando actividades con mi familia (caminando, jugando).	I spend time carrying out activities with my family (walking, playing).
3	Voy a ver a la enfermera o al médico del colegio si no me estoy sintiendo bien.	I go see the school nurse or doctor if I am not feeling well.
4	Realizo actividad física vigorosa por 20 minutos o más, 3 veces a la semana (danza aeróbica, caminar enérgicamente, correr, saltar lazo montar en bicicleta, nadar).	I engage in vigorous physical activity for 20 minutes or more, three times per week (aerobic dancing, brisk walking, running, jumping rope, cycling, swimming).
5	Duermo entre 6 y 8 horas en la noche.	I sleep between 6 and 8 hours at night.
6	Felicito a los demás cuando hacen algo bien.	I congratulate others when they do something well.
7	Evito los dulces u otros alimentos ricos en azúcar.	I avoid sweets and other foods high in sugar.
8	Leo artículos sobre temas de salud.	I read articles about health topics.
9	Hablo con los demás sobre mis creencias espirituales.	I talk with others about my spiritual beliefs.
10	Elijo leche o productos lácteos bajos en grasa (yogurt, queso, helado).	I choose milk or low-fat dairy products (yogurt, cheese, ice cream).
11	Dedico tiempo cada día para descansar.	I take time each day to rest.
12	Procuro ser sensible frente a los sentimientos de los demás.	I try to be sensitive to the feelings of others.
13	Desayuno.	I have breakfast.
14	Hago preguntas al doctor o a la nurse para entender sus instrucciones.	I ask questions to the doctor or nurse to understand their instructions.
15	Siento que hay un poder superior guiando mi vida.	I feel there is a higher power guiding my life.
16	Participo en actividades recreativas o deportes.	I participate in recreational or sports activities.
17	Acepto las cosas de mi vida que no puedo cambiar.	I accept the things in my life that I cannot change.
18	Me siento entusiasmado sobre el futuro.	I feel excited about the future.
19	Paso tiempo con amigos cercanos.	I spend time with close friends.
20	Asisto a un grupo que comparte mis creencias espirituales.	I attend a group that shares my spiritual beliefs.
21	Como entre 2 y 4 porciones de fruta al día.	I eat between 2 and 4 portions of fruit per day.
22	Asisto a programas de prevención y mejoramiento de la salud.	I attend health prevention and improvement programs.
23	Estoy feliz con quien soy.	I am happy with who I am.
24	Como entre 3 y 5 porciones de verduras cada día.	I eat between 3 and 5 portions of vegetables per day.
25	Saco tiempo para hacer las cosas que me gustan.	I make time to do the things I like.
26	Trabajo para alcanzar metas importantes en mi vida.	I work to reach important goals in my life.
27	Camino o realizo alguna actividad en mi tiempo libre.	I walk or conduct some activity in my free time.
28	Espero con ansias cada nuevo día.	I look forward anxiously to each new day.
29	Realizo actividades que me ayudan a crecer espiritualmente.	I carry out activities that help me to grow spiritually.
30	Como variedad de carnes (pollo, pescado, res, cerdo).	I eat a variety of meats (poultry, fish, beef, pork)
31	Resuelvo mis conflictos a través del diálogo en lugar de la pelea.	I settle my conflicts through dialogue rather than fighting.
32	Juego juegos activos con mis amigos (baloncesto, softbol, voleibol, tenis, etc.).	I play active games with my friends (basketball, softball,volleyball, tennis, etc.,).
33	Busco la guía del consejero escolar cuando la necesito.	I seek guidance from the school counselor when I need it.
34	Consulto al doctor o a la enfermera sobre cómo mejorar mi salud.	I consult with the doctor or nurse about how to improve my health.
35	Dedico tiempo a la oración o a la meditación.	I dedicate time to prayer and meditation.
36	Trato de tener pensamientos agradables mientras me quedo dormido.	I try to have pleasant thoughts while I fall asleep.
37	Hago un esfuerzo especial por ser útil a los demás.	I make a special effort to be useful to others.
38	Me fijo metas que puedo alcanzar.	I set goals I can reach.
39	Me siento bien conmigo mismo cuando hago algo bien.	I feel good with myself when I do something well.
40	Hago ejercicio hasta que se me acelera el corazón and sudo.	I exercise until my heart races and I sweat.
41	Uso mis creencias espirituales como guía para lo que hago.	I use my spiritual beliefs as a guide for what I do.
42	Bebo seis (6) o más vasos de agua al día.	I drink six (6) or more glasses of water per day.
43	Discuto mis problemas con alguien cercano para intentar resolverlos.	I discuss my problems with someone close to try to solve them.
44	Evito comportamientos perjudiciales para mi salud (fumar, consumir alcohol, consumir drogas, actividades sexuales)	I avoid behaviors that are harmful to my health (smoking, alcohol consumption, drug use, sexual activities).
